# Probing time-dependent mechanical behaviors of catch bonds based on two-state models

**DOI:** 10.1038/srep07868

**Published:** 2015-01-19

**Authors:** Xiaofeng Chen, Zhixiu Mao, Bin Chen

**Affiliations:** 1 Department of Engineering Mechanics, Zhejiang University, Hangzhou 310027, P. R. China

## Abstract

With lifetime counter-intuitively being prolonged under forces, catch bonds can play critical roles in various sub-cellular processes. By adopting different “catching” strategies within the framework of two-state models, we construct two types of catch bonds that have a similar force-lifetime profile upon a constant force-clamp load. However, when a single catch bond of either type is subjected to varied forces, we find that they can behave very differently in both force history dependence and bond strength. We further find that a cluster of catch bonds of either type generally becomes unstable when subjected to a periodically oscillating force, which is consistent with experimental results. These results provide important insights into versatile time-dependent mechanical behaviors of catch bonds. We suggest that it is necessary to further differentiate those bonds that are all phenomenologically referred to as “Catch bonds”.

Physical interactions between proteins are often mediated by weak but specific adhesive bonds, functioning through a lock-and-key mechanism^1^,^2^. The dissociation of specific bonds can be regulated by forces, which is often regarded as thermally assisted escape over an energy barrier^1^,^2^. Based on the dependence of the dissociation rates on forces, there exist three categories of bonds, including ideal bonds, whose dissociation rates are independent of forces, slip bonds, whose dissociation rates increase as forces increase, and catch bonds, whose dissociation rates counter-intuitively decrease and then eventually increase with forces.

Dembo et al.^3^ originally proposed catch bonds in their theoretical consideration of reversible adhesion upon forces. Though quite a few experiments^4^,^5^,^6^ were in consistency with the presense of catch bonds, alternative interpretations could not be rigorously ruled out. Combining molecular dynamics simulation and mutagenesis analysis, Thomas et al.^7^ provided evidence for catch bonds formed between E. coli fimbrial adhesin FimH receptors and mannose ligands. With atomic force microscopy, Marshall et al.^8^ found that increasing forces prolonged and then eventually shortened the lifetime of P-selectin complexes with P-selectin glycoprotein ligand-1 (PSGL-1), which provided a definitive proof of catch bonds. By now, various protein interactions were demonstrated to exhibit catch bond behavior in experiments^8^,^9^,^10^,^11^,^12^,^13^,^14^,^15^. One common function of these revealed catch bonds is to support or transmit forces^16^. Catch bonds provide a critical way to stabilize attachments exactly when needed^17^, which play critical roles in various sub-cellular processes.

Structural basis varies for different catch bonds. For example, catch bonds are formed between the E. coli fimbrial adhesin FimH receptor and mannose. In the absence of mannose, the mannose-binding domain in fimbrial tips is of a loose mannose-binding pocket due to allosteric auto-inhibition^18^. When mannose binds, the pocket can tighten around mannose and some newly-formed bonds can be immediately at a strong adhesive state of an elongated conformation. If these bonds are subjected to forces before mannose dissociates, the elongated conformation may be stabilized, leading to longer bond lifetime under force. In comparison, for catch bonds formed between P-selectin and PSGL-1, molecular dynamics simulations suggested that the newly formed bond is initially at a weak adhesive state without forces^19^. With forces, an interdomain hinge can open and the binding interface would be tilted to allow contacting surfaces to slide against each other, thereby slowing dissociation and prolonging bond lifetime. Thus, forces prevent bonds formed between FimH and mannose from switching from an initial strong adhesive state to a weak adhesive state, but facilitate bonds formed between P-selectin and PSGL-1 to switch from an initial weak adhesive state to a strong adhesive state. These two different “catching” strategies both lead to catch bonds.

Most recently, intriguing observations on the time-dependent behaviors of catch bonds were reported. For example, upon a constant force-clamp load, the bonds between L-selectin and PSGL-1 behaved as catch bonds at low ramp rates but as slip bonds at very high ramp rates (>7000 pN/s)^20^. It was reported that catch bonds formed between fibronectin and integrin *α*_5_*β*_1_ switched from a weak adhesive state to a strong adhesive state upon specific cyclic forces^21^. The bond lifetime was surprisingly prolonged up to two orders of magnitude, when the bond was firstly loaded to a large force and then unloaded to and clamped at a low force^21^. It was also reported that the bond lifetime increased with cycles upon cyclic forces until saturated beyond a few cycles^21^. This phenomenon was termed as “Cyclic mechanical reinforcement”^21^.

These time-dependent mechanical behaviors of catch bonds can be very important and closely related to their biological functions, though studies of them appear to be limited. Here we mathematically formulate two types of catch bonds with two different “catching” strategies within the framework of two-state models^17^. We then investigate their behaviors by applying varied loads to both types of catch bonds. Such a quantitative approach should be very useful to predict the behaviors of bonds in new situations^17^. In the following, we firstly show that a single catch bond of either type can manifest with a similar force-lifetime profile upon a constant force-clamp load. We then apply varied forces to either a single catch bond or a cluster of catch bonds. The subsequent analysis indicates that time-dependent behaviors of catch bonds can be versatile. This work suggests that it is necessary to further differentiate those bonds that are all phenomenologically referred to as “Catch bonds”.

## Two types of catch bonds constructed with different “catching” strategies based on two-state models

We let an associated bond have two adhesive states, a relatively strong adhesive state and a relatively weak adhesive state. Two types of catch bonds are then constructed based on two different “catching” strategies. For the first type (Type I), a newly formed bond is assumed to be at the weak state initially and the applied force can facilitate its switching to the strong state. In contrast, a newly formed bond is assumed to be initially at the strong adhesive state for the second type (Type II) and the applied force can prevent it from switching to the weak state. Since forces can facilitate the bond formed between P-selectin and PSGL-1 to switch from an initial weak adhesive state to a strong adhesive state, such a bond can belong to Type I bonds. On the other hand, since forces can prevent the bond formed between FimH and mannose from switching from an initial strong adhesive state to a weak adhesive state, such a bond can belong to Type II bonds.

**Figure 1:**
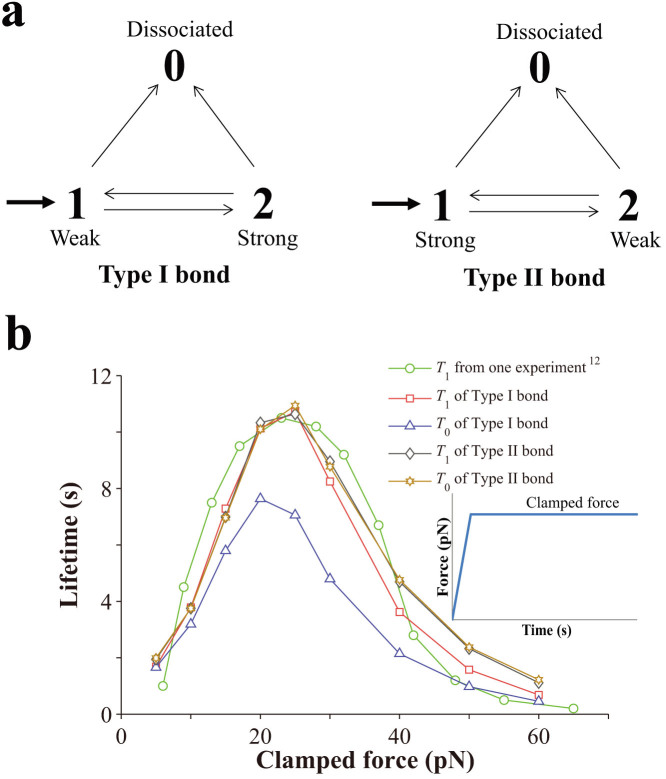
(a) Two types of catch bonds constructed with different “catching” strategies. A newly formed bond is at State 1 initially, which can then switch back and forth between State 1 and State 2 or dissociate from either State 1 or State 2 to State 0. Note that State 1 is a weak adhesive state for Type I catch bond, while State 1 is a strong adhesive state for Type II catch bond. An applied force can facilitate the switching to the strong state for Type I bond while preventing it from switching to the weak state for Type II bond. (b) Variation of the lifetime of a single catch bond with the clamped force. Inset is the profile of a constant force-clamp load.

The two-state models for both types of bonds are provided in Fig. 1(a), where a dissociated bond is assigned with State 0. In the model, an associated bond can switch back and forth between two adhesive states and dissociate from either state.

The dependence of all transition rates among states on the applied force is described by the Bell's law^22^. A newly formed bond is initially at State 1. The transition rate from State 1 to State 0 is given by 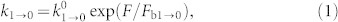
where 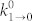 is the transition rate without force and *F*_b1→0_ is a force scale. The transition rate from State 2 to State 0 is given by 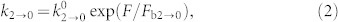
where 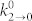 is the transition rate without force and *F*_b2→0_ is a force scale. The transition rate from State 1 to State 2 is given by 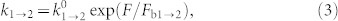
where 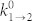 is the transition rate without force and *F*_b1→2_ is a force scale. The transition rate from State 2 to State 1 is given by 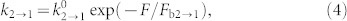
where 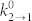 is the transition rate without force and *F*_b2→1_ is a force scale.

We then apply a constant force-clamp load to a newly formed bond where the load increases linearly with time at a ramp rate of 500 pN/s until it is clamped at a certain value, as schematically shown in the inset of Fig. 1(b). Kinetic Monte Carlo method is employed to simulate the lifetime of a single bond against the clamped load^23^. For more details of the simulation, please refer to the Supplementary Information. Sometimes, a bond is already dissociated before the applied force reaches the clamped value in the simulation. When these short-lived events are excluded in averaging lifetime and timing starts from the time exactly when the bond is clamped and continues until the bond is dissociated, as usually done in experiments^12^,^20^,^21^, the corresponding lifetime is denoted as *T*_1_. In contrast, if these short-lived events are included in averaging lifetime and timing starts from the time exactly when the force is applied and continues until the bond is dissociated, it is denoted as *T*_0_.

Generally, the transition rates can range from 0.01 Hz to a few Hzs, and the force scales can be around a few pNs^2^ and up to ~20 pN^24^. Designed parameters we have chosen for Type I bonds are listed in Table 1, while those for Type II bonds are listed in Table 2. As inferred from these parameters, State 1 is indeed much weaker than State 2 for Type I bonds, while State 1 is indeed much stronger than State 2 for Type II bonds. In choosing these parameters, we consider those that are physically reasonable and can also fit the experimental data^12^.

With these parameters, we loop the simulation for 10000 times for each force to get the mean lifetime, *T*_1_ and *T*_0_, for both types of bonds and the error of the mean lifetime is 1%. As seen from the profile of lifetime against clamped force in Fig. 1(b), both types of bonds manifest with a similar catch-bond behavior and there is trivial difference in *T*_1_ of both types of bonds. A set of experimental data of *T*_1_^12^ are also plotted out in Fig. 1(b), which are close to our simulated *T*_1_ values. It should be pointed out that our designed parameters are not unique in fitting the experimental data^12^. We have investigated the sensitivity of these parameters by slightly varying them in the simulations and find that, though the magnitude of *T*_1_ or *T*_0_ would change a little, overall trends maintain.

With these two types of catch bonds constructed here, we further probe their time-dependent mechanical behaviors in the following.

## Analysis and discussions

### Force history dependence

**Figure 2:**
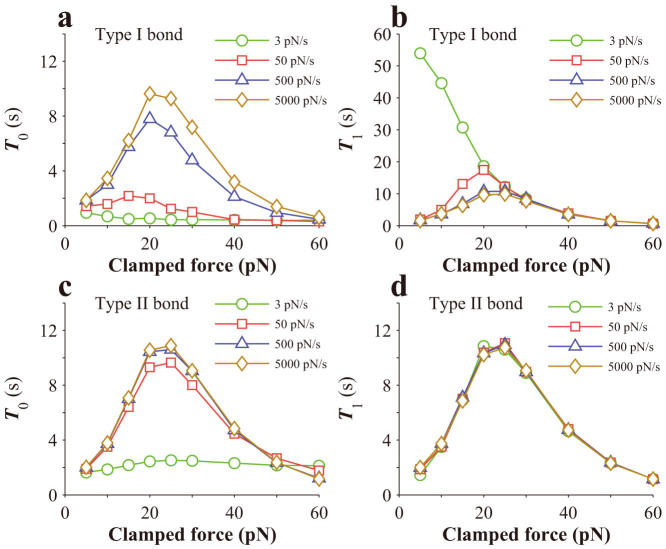
Effects of ramp rates on the lifetime of a single catch bond when a constant force-clamp load is applied. (a,b) *T*_0_ and *T*_1_ of Type I bonds. (c,d) *T*_0_ and *T*_1_ of Type II bonds.

When an associated bond can switch among different adhesive states with the corresponding switching rates depending on forces, its lifetime may depend on its loading history. To investigate such a memory-like effect, we firstly apply a constant force-clamp load to a single bond and investigate the effects of ramp rates, as shown in Fig. 2. As seen in Fig. 2b, ramp rates have a strong effect on *T*_1_ of Type I bonds and decreasing the ramp rates significantly increases the bond lifetime *T*_1_ at small clamped force (<20 pN). Most interestingly, according to Fig. 2b, Type I bonds manifest as slip bonds at very low ramp rates, for example, at 3pN/s. In contrast, ramp rates have an almost trivial effect on *T*_1_ of Type II bonds, as seen in Fig. 2d. The different effects of ramp rates on *T*_1_ of two types of bonds are due to the pre-selection during the ramping phase. For Type I bonds that have survived through the ramping phase at low ramping rates, a large portion have switched from State 1 (the weak state) to State 2 (the strong state) during the ramping phase. Since it is not easy to switch from State 2 back to State 1, Type I bond behaves more like a slip bond at the strong state under this condition, as indicated in Fig. 2b. For Type II bonds that have survived through the ramping phase at low ramping rates, most of them remain in State 1, which is the strong state. These bonds can later switch to State 2 depending on the clamped forces and still manifest as catch bonds, as indicated in Fig. 2d. We also plot out *T*_0_ of both types of bonds in Figs. 2a,c, respectively. As seen from Fig. 2a, ramp rates also have a strong effect on *T*_0_ of Type I bonds. However, decreasing the ramp rates decreases *T*_0_, which is of an opposite trend to that of *T*_1_. As seen from Fig. 2c, ramp rates also have a strong effect on *T*_0_ of Type II bonds and it manifests almost as ideal bonds at a ramp rate of 3pN/s, where lifetime varies little with the clamped forces.

We emphasize that effects of ramp rates on *T*_1_ of Type I bonds appear to be different from a recent experimental report on force history dependence of catch bonds^20^. It was shown that the bond formed between L-selectin and PSGL-1 behaved as a catch bond at low ramp rates but as slip bonds at very high rates (>7000 pN/s)^20^. It was further shown that the effect of ramp rates on bond lifetime was similar to that of a point mutation at the L-selectin surface, which could be explained by a modified sliding-rebinding model where the initial adhesive state depended on the ramp rate^20^. Thus, our findings may complement the recent experimental report on force history dependence of catch bonds^20^ and suggest that the structural basis for the force history dependence of bond lifetime can be versatile.

The lifetime of the catch bond^12^ formed between fibronectin and integrin *α*_5_*β*_1_ was also found to depend on force history. It was revealed that this bond switched from a weak adhesive state to a strong adhesive state upon specific cyclic forces^21^. The bond lifetime was surprisingly prolonged up to two orders of magnitude, when the bond was firstly loaded to a large force and then unloaded before it was clamped at a low force^21^. It was also reported that the bond lifetime increased with cycles upon cyclic forces until saturated beyond a few cycles^21^. Such an interesting phenomenon of force history dependence was also termed as “Cyclic mechanical reinforcement”^21^.

**Figure 3:**
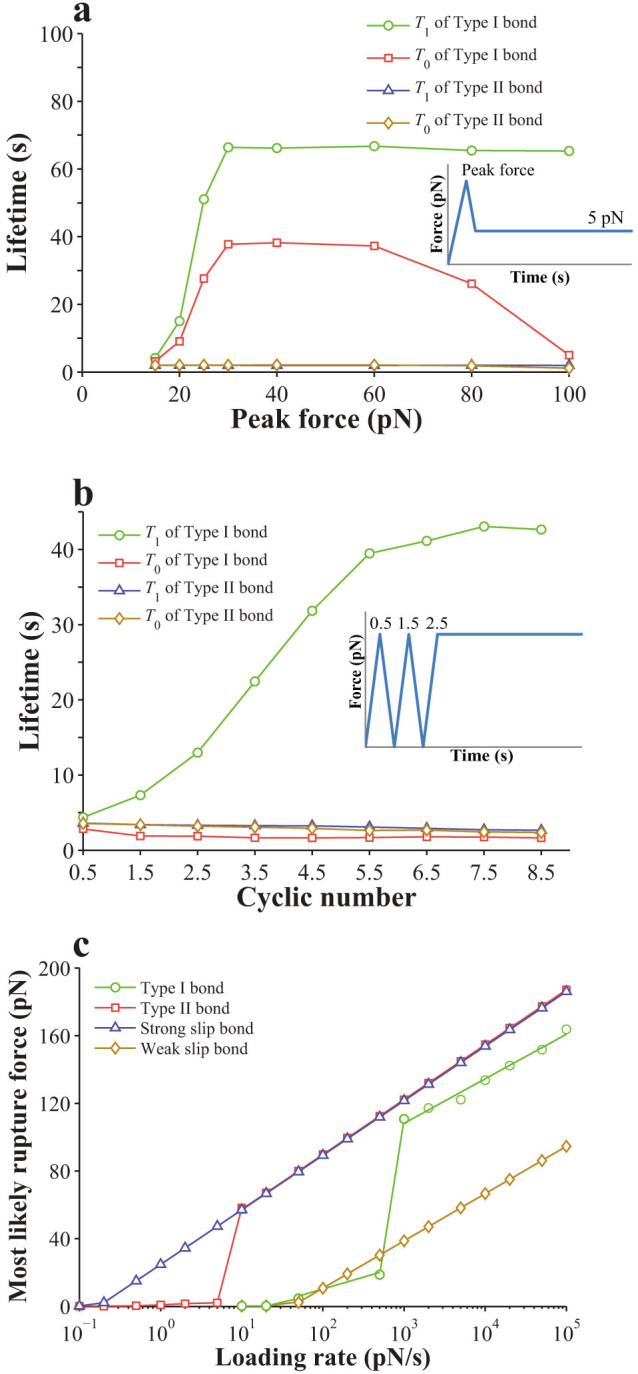
Force history dependence of bond lifetime. (a) Variation of lifetime of a single catch bond with the peak force. Inset is the profile of the applied force. (b) Effects of cyclic number on the lifetime of a single catch bond. Inset is the profile of the applied force. (c) Variation of bond strength with loading rates upon a steady force-ramp load. The dissociation rate of the strong slip bond is given by 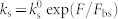, where 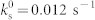 and *F*_bs_ = 14.0 pN, and that of the weak slip bond is given by 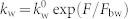, where 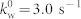 and *F*_bw_ = 12.0 pN.

We then investigate whether these two types of bonds would manifest with “Cyclic mechanical reinforcement”. In one case of our analysis, a bond is loaded to a peak force and then unloaded before it is clamped at 5pN, as shown in the inset of Fig. 3a, and the dependence of bond lifetime on the peak force is investigated. The loading and unloading rates are 500 pN/s and −500 pN/s, respectively. As shown in Fig. 3a, Type I catch bonds clearly manifest with reinforcement, while Type II catch bonds do not. As also seen in Fig. 3a, *T*_1_ of Type I bonds increases with the peak force at first and then saturates beyond a certain value, while *T*_0_ of Type I bonds increases with the peak force at first and then decreases beyond a certain value.

In another case of our analysis, a bond is loaded to a peak force and then completely unloaded, which is repeated for several times before it is clamped at the peak force, as schematically shown in the inset of Fig. 3b. We investigate the effect of cyclic number on the bond lifetime. In the simulation, the peak force is 10 pN, the ramp rate for the loading phase is 100 pN/s, and that for the unloading phase is −100 pN/s, similar to loading conditions in experiments^21^. As seen from Fig. 3b, *T*_1_ of Type I bonds dramatically increases with cyclic number until it saturates. In contrast, *T*_1_ of Type II bond is rather insensitive to cyclic number. We also simulate *T*_0_ for both types of bonds. As seen from Fig. 3b, *T*_0_ of Type II bond appears to be insensitive to cyclic number, either. However, *T*_0_ of Type I bonds slightly decreases with the cyclic number, which can be due to the relatively small peak force adopted in the simulation, making bonds hard to switch from State 1 to State 2. When increasing the peak force in the simulation, we find that *T*_0_ of Type I bonds increases instead. Putting these results together, our simulations indicate that only *T*_1_ of Type I bonds manifests with strong “Cyclic mechanical reinforcement”. However, since it is unclear whether *T*_0_ or *T*_1_ is more relevant to cell adhesion, it should be cautioned that “Cyclic mechanical reinforcement” must be carefully interpreted in accounting for strengthening cell adhesion upon cyclic forces.

In understanding the catch-bond behavior of PSGL-1-P-selection bond, Evans et al.^23^ also employed a two-state model by assuming two possible bound states that remained thermally equilibrated with the energy difference between them shifting in proportion to force. The model by Evans et al.^24^ quantified the switching between two bound states and its prediction of bond strength and force histograms was in agreement with the experiment^24^. However, the possibility of the two bound states in Evans et al.^24^, as well as the bond dissociation rate^25^, depended only on the immediate force, instead of the force history. In other words, the bond would not switch efficiently from a weak adhesive state to a strong adhesive state upon specific cyclic forces in the modeling framework of Evans et al.^24^, which would be inconsistent with the experiments^21^.

### Effects of loading rates on bond strength

We then investigate effects of loading rates on bond strength, i.e., the most likely rupture force, when a bond is subjected to a steady force-ramp load. One key feature of slip bonds is that the bond strength increases almost linearly with the logarithm of the loading rate. For example, when the breaking rate of a slip bond is given by 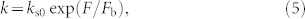
where *k_s_*_0_ is the breaking rate without force and *F*_b_ is a force scale, the dependence of bond strength, f*, on the loading rate, *r_f_*, will be given by 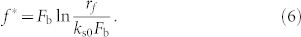
Since f* must be positive*,* Eq. (6) is valid only when *r_f_* is above a loading rate of *k_s_*_0_*F*_b_.

As seen from Fig. 3c, such a linear dependence does exist for slip bonds when the regime of very low loading rates is excluded. However, there appears to be three different regimes in the dependence of bond strength on loading rates for both Type I bonds and Type II bonds. Prominently, there exists a jump in the bond strength around a critical loading rate. Such a jump occurs at a very small loading rate, ~6 pN/s, for Type II bond, above which bond strength increases almost linearly with the logarithm of the loading rate. In comparison, such a jump occurs at a much higher loading rate for Type I bond, ~600 pN/s, which is about two orders of magnitude higher. Such a jump in bond strength was also predicted by Evans et al.^24^.

**Figure 4:**
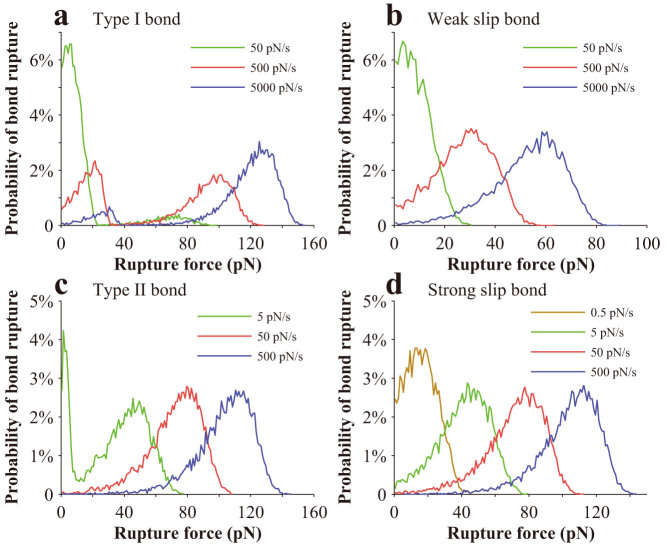
Variation of the probability of bond rupture with rupture force when a bond is subjected to a steady force-ramp load. (a) Type I bond. (b) Weak slip bond. (c) Type II bond. (d) Strong slip bond. The dissociation rate of the strong slip bond is given by 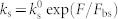, where 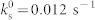 and *F*_bs_ = 14.0 pN, and that of the weak slip bond is given by 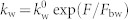, where 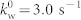 and *F*_bw_ = 12.0 pN.

We also plot out the probability of bond rupture versus force for slip bonds, Type I catch bonds, and Type II catch bonds at different loading rates, as shown in Fig. 4. For both strong slip bonds and weak slip bonds, there exists only one peak in the probability curves. For Type II catch bonds, there also exists only one peak at high loading rates and the curves gradually shift rightward as the loading rate increases, which is very similar to that of slip bonds. However, at low loading rates, for example, at 5pN/s, there exist two peaks. Differently, there exist two peaks at all loading rates under our investigation for Type I bonds.

These results might be relevant to a puzzling issue that different experimental procedures had provided contradicting results of bond types^8^,^9^,^26^,^27^,^28^. When a constant force-clamp load was applied and lifetime versus force was measured, catch bonds were observed^8^,^9^,^28^. However, when a steady force-ramp load was applied and most likely rupture force versus loading rate was measured, only slip bonds were observed for the same interactions^26^,^27^. Such contradicting results were reported in various interactions, including bonds formed between ligands and P-selectin, L-selectin, E-selectin, etc^8^,^9^,^26^,^27^,^28^. Interestingly, our studies here suggest that those observed catch bonds might be similar to Type II catch bonds, which behave like slip bonds when a steady force-ramp load is applied at relatively high loading rates.

### Instability upon periodically oscillating forces

Periodically oscillating forces regulate cellular structures and functions under physiological conditions^29^. It was clearly demonstrated that polarized cells on elastic substrates aligned themselves almost perpendicular to the loading direction upon uni-axial cyclic stretches^30^. It was recently proposed that cyclic stretch can induce cell reorientation on substrates by destabilizing catch bonds in focal adhesions^31^. Here, we investigate whether a periodically oscillating force would destabilize two types of catch bonds at the level of a single bond or a cluster.

**Figure 5:**
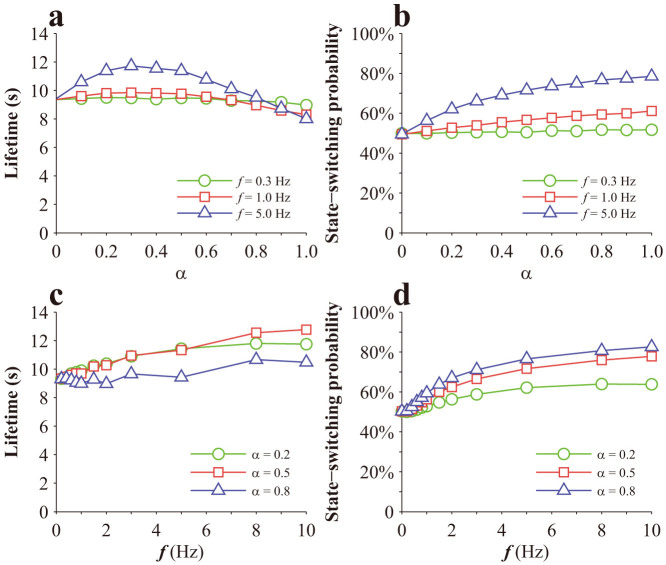
Effects of a periodically oscillating load on a single Type I catch bond: (a) Variation of lifetime with stretch amplitude; (b) Variation of state-switching probability from State 1 to State 2 with stretch amplitude; (c) Variation of lifetime with cyclic frequency; (d) Variation of state-switching probability from State 1 to State 2 with cyclic frequency.

**Figure 6:**
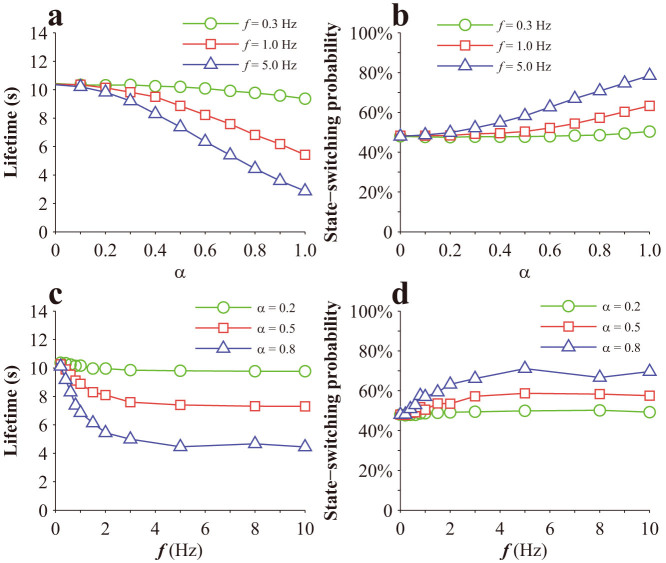
Effects of a periodically oscillating load on a single Type II catch bond: (a) Variation of lifetime with stretch amplitude; (b) Variation of state-switching probability from State 1 to State 2 with stretch amplitude; (c) Variation of lifetime with cyclic frequency; (d) Variation of state-switching probability from State 1 to State 2 with cyclic frequency.

According to Chen et al.^31^, one form of the oscillating force is given by 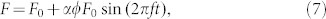
where *F*_0_ is the average value of *F* within one period related to the homeostatic tension within focal adhesions (FAs), *f* is the cyclic frequency, *t* is the time, *α* is the stretch amplitude, and *φ* reflects the regulation of the contractile stress fiber (SF) on the applied force, given by^31^

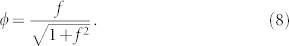
Note that, in calculating the results in Fig. 3b, a cyclic force is applied, as schematically shown in the inset of Fig. 3b. That cyclic force is different from the oscillating force here, which oscillates around a homeostatic value according to Eqs. (7,8). Monte Carlo method is employed to simulate the lifetime of a single bond. In the simulation, *F*_0_ = 20 pN. The results for a single Type I bond are given in Fig. 5. As seen from Figs. 5a,c, there is generally no monotonic dependence of lifetime on stretch amplitude or cyclic frequency. However, there appears to be a biphasic dependence of bond lifetime on the stretch amplitude at relatively high cyclic frequencies, as shown in Fig. 5a. The probability of Type I bond switching from State 1 to State 2 generally increases with both stretch amplitude and cyclic frequencies, as shown in Figs. 5b,d. The results for a single Type II bond are given in Fig. 6. Its lifetime generally decreases with stretch amplitude, as shown in Fig. 6a, and also decreases with cyclic frequencies until a certain value, as shown in Fig. 6c, which is consistent with a previous prediction^31^. The probability of Type II bond switching from State 1 to State 2 generally increases with both stretch amplitude and cyclic frequencies, as shown in Figs. 6b,d.

It appears that, when the applied force oscillates around a homeostatic value, the unloading phase may be more important in determining the switching probability from State 1 to State 2 for Type II bond. Larger stretch amplitude would result in lower force values in the unloading phase, which would be less efficient in preventing the bond switching from State 1 to State 2, in consistency with Fig. 6d. In contrast, when the applied force oscillates around a homeostatic value, the loading phase may be more important in determining the switching probability from State 1 to State 2 for Type I bond. Larger stretch amplitude would result in higher force values in the loading phase, which would be more efficient in facilitating the bond switching from State 1 to State 2. This would then be in consistency with our simulation results in Fig. 6b.

It should be noted that, in the study of a single bond, bond rebinding is not considered in the current work. However, bond rebinding may not be prevented in the experiments when the loading rate is very low. With the consideration of this rebinding, Li and Ji^32^ predicted a nontrivial bond strength at very low loading rates, which differs from the conventional wisdom but is in excellent consistency with the experiments. As indicated in Fig. 3c, without rebinding, the bond strength for either type of catch bond vanishes at a very low loading rate, similar to a slip bond. We suspect that either type of catch bond may also manifest with nontrivial bond strength at very low loading rates when bond rebinding is considered. We would rather leave such a study for our future investigation.

**Figure 7:**
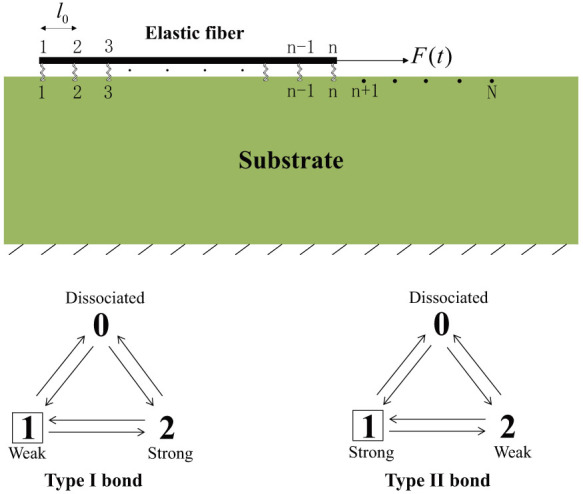
An elastic fiber is adhered to a rigid substrate through a cluster of catch bonds and subjected to a periodically oscillating force, *F*(*t*), on its right end. The neighboring distance of receptors and ligands is *l*_0_. The total number of receptors and ligands are n and N, respectively. The fiber is modeled as 1-d elastic rod and the bonds as elastic springs. The bonds within the cluster are at State 1 initially and it is assumed that the state information of a bond is contained within the receptor only and the initial state of a re-formed bond is the same as that of the receptor right before it is broken.

Within focal adhesions, molecular bonds often function as a cluster. The lifetime of a cluster of catch bonds is simulated with a coupled finite element analysis and Monte Carlo method^23^,^33^,^34^. As schematically shown in Fig. 7, the cluster adheres an elastic fiber with tensional modulus, *EA*, to a rigid substrate. Receptors and ligands are uniformly distributed along the fiber and on a portion of substrate surface, respectively, with neighboring distance, *l*_0_ = 32 nm^33^. The total number of receptors is n = 40 and that of ligands is N = 160. Initially, all receptors form closed bonds with ligands and the formed bonds are all at State 1, which can then switch back and forth between State 1 and State 2 and can also randomly break from either state. Broken receptors can randomly rebind to free ligands. It is assumed that the state information of a bond is contained within the receptor only and the initial state of a re-formed bond is the same as that of the receptor right before it is broken.

The bond rebinding rate is given by^23^

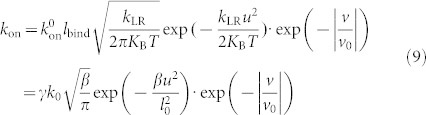
where 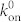 is the reaction rate when the ligand and its receptor are within a small binding distance, *l*_bind_^35^, *K_B_* is Boltzmann constant, *T* is the temperature, *k*_LR_ is the spring constant of the formed bond, *u* is the distance between the ligand and its receptor, *v* is the relative velocity between a ligand and its receptor, *ν*_0_ = *a*/*τ*_0_ is an intrinsic velocity^36^, with *a* being 10 nm and *τ*_0_ being an intrinsic association time ranging from 0.01 s to 1 s^37^,^38^, *k*_0_ is a rate constant, 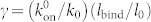, and 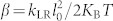. In the simulation, *F*_0_ = 400 pN, *EA* = 46*nN*^39^, *k*_LR_ = 0.25 pN/nm^33^, *v*_0_ = 400 nm/s^36^, *γ* = 10^31^, and *k*_0_ = 0.1/s^33^. The simulation proceeds until all receptors are open and the corresponding time duration is taken as the lifetime of the bond cluster. The mean lifetime of the bond cluster is obtained by running the simulation for 100 times and its error is 10%.

**Figure 8:**
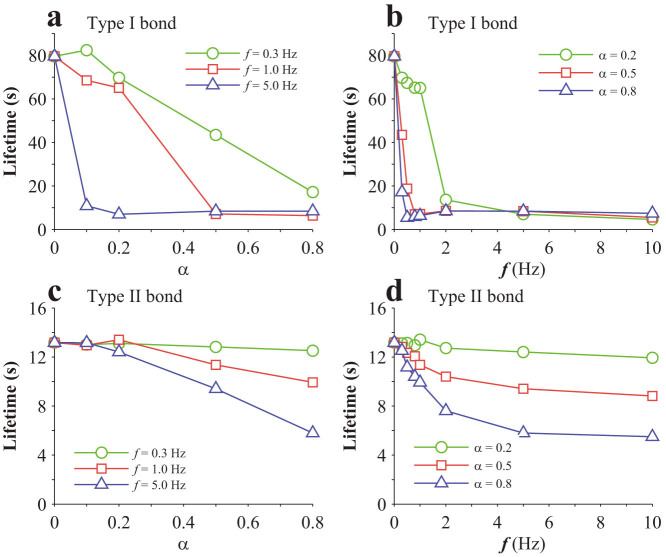
Effects of periodically oscillating forces on a bond cluster: (a,b) Variation of lifetime with stretch amplitude or cyclic frequencies for Type I catch bonds; (c,d) Variation of lifetime with stretch amplitude or cyclic frequencies for Type II catch bonds.

The effect of stretch amplitude or cyclic frequency on the lifetime of the bond cluster is shown in Fig. 8, which indicates that a bond cluster generally becomes unstable when subjected to a periodically oscillating force. As seen in Figs. 8a,c, the cluster of Type I bonds appears to be more stable than that of Type II bonds at the same stretch amplitude, though both of their lifetimes generally decrease as stretch amplitude increases. As seen in Figs. 8b,d, the cluster of Type I bonds appears to be more stable than that of Type II bonds at the same cyclic frequency, though both of their lifetimes generally decrease as cyclic frequency increases until saturate beyond a certain value, which seems to be consistent with the experiments^30^.

As indicated in Fig. 5, certain stretch amplitude and cyclic frequencies of oscillating force prolongs the lifetime of Type I catch bond. However, Fig. 8 indicates such oscillating force reduces the lifetime of the bond cluster formed by Type I catch bond. This inconsistence is due to rebinding of broken bonds occurring within the bond cluster, which apparently have a significant effect on the lifetime of the cluster. According to Eq. (9), the rebinding rate of a broken bond within the cluster depends on *v*, which is the relative velocity between a ligand and its receptor. A larger *v* would lead to a lower rebinding probability of a broken bond. Note that a larger stretch amplitude or a higher cyclic frequency would induce a larger *v*. The rebinding probability of broken bonds would then be lower under this condition, which may explain why the lifetime of the bond cluster formed by Type I catch bond can decrease with both stretch amplitude and cyclic frequency in Fig. 8.

In the end, we should emphasize that the initial state of bonds in our study of a single catch bond or a cluster of catch bonds is assumed to be 100% at State 1 in developing the theory in the current work. Though this assumption might be valid under physiological conditions in some cases, it may be not true in some experiments. For example, ~2% bonds were predicted to be initially at State 2 in Evans et al.^24^. This is because a finite duration of time is generally required to prompt bond formation between receptors and ligands before the force is applied in experiments. Within this duration, the switching of bond states may have taken place, which should depend on zero-force kinetics of bond formation, transition between the two states, as well as the duration. How an initial distribution of bond states quantitatively affects our theory prediction will be addressed in the future work.

## Conclusion

We formulate two types of catch bonds based on different “catching” strategies within the framework of two-state models. For Type I, a newly formed bond is assumed to be in the weak state initially and the applied force can facilitate its switching to the strong state. For Type II, a newly formed bond is assumed to be in the strong adhesive state initially and the applied force can prevent it from switching to the weak state. With chosen parameters, both types of catch bonds manifest with a similar force-lifetime profile upon a constant force-clamp load. We then show that ramp rates of a constant force-clamp load have a strong effect on *T*_1_ of Type I bonds but an almost trivial effect on *T*_1_ of Type II bonds. We also show that only *T*_1_ of Type I bonds manifests with strong “Cyclic mechanical reinforcement” while that of Type II bonds does not. We further show that there exists a jump at a critical loading rate in the bond strength for both types of catch bonds, above which bond strength increases almost linearly with the logarithm of the loading rate; however, the critical loading rate at which the jump occurs for Type II bonds is much lower than that for Type I bonds. We also find that a cluster of both Type I bonds and Type II bonds generally become unstable when subjected to a periodically oscillating force, which is consistent with experimental results^29^. These findings provide important insights into understanding time-dependent mechanical behaviors of catch bonds^20^,^21^,^24^,^40^. Based on these results, we propose to further differentiate those bonds that are all phenomenologically referred to as “catch bonds”.

In the end, we should emphasize that it would be desirable to identify specific structural basis in a particular catch bond system to verify quantitative theories as developed in this work in the future, though such theories are useful for predicting the behaviors of bonds in new situations^17^. Such an approach is promising through characterizing crystal structures of receptors with or without binding with ligands^18^, analysis of protein sequences, and molecular dynamics simulations^19^,^41^.

## Methods

Kinetic Monte Carlo method is employed for the simulation of a single catch bond with the details given in the Supplementary Information. For a cluster of catch bonds, a coupled Finite Element method and Monte Carlo method is employed with the detailed simulation procedure provided in our previous work^23^.

## Author Contributions

B.C. designed the work, X.C. performed the simulations, X.C. and Z.M. analyzed the data, and X.C. and B.C. wrote the manuscript. All authors reviewed the manuscript.

## Supplementary Material

Supplementary Information Supplementary Information

## Figures and Tables

**Table 1:** Designed parameters for Type I bond

Transition rate	Value (s^−1^)	Force scale	Value (pN)
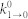	3.0	*F*_b1→0_	12.0
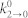	0.01	*F*_b2→0_	12.0
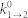	0.02	*F*_b1→2_	3.0
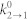	0.001	*F*_b2→1_	1.0

**Table 2:** Designed parameters for Type II bond

Transition rate	Value (s^−1^)	Force scale	Value (pN)
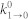	0.012	*F*_b1→0_	14.0
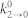	1.0	*F*_b2→0_	4.0
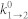	1.3	*F*_b1→2_	−6.0
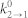	0.001	*F*_b2→1_	1.0
