# Supplementary material for: Probing time-dependent mechanical behaviors of catch bonds based on two-state models
Source: Sci Rep. 2015 Jan 19;5:7868. doi: 10.1038/srep07868 (PMC4297987; doi:10.1038/srep07868)
Supplement: Supplementary Information [file srep07868-s1.pdf]

Supplementary Information

Title: Probing time-dependent mechanical behaviors of catch bonds based on two-state models

Authors: Xiaofeng Chen, Zhixiu Mao, Bin Chen

Kinetic Monte Carlo simulation for a single catch bond

A single catch bond is initially at State 1, which can then switch back and forth between State 1 and State 2 or be dissociated from either state. Kinetic Monte Carlo method is employed to determine when and where the next random event would occur. Let $a_i\left(\mathrm{i}=1,2\right)$ denote the switching rates, $k_{1\to2}$ and $k_{1\to0}$, if the bond is currently at State 1 or , $k_{2\to1}$ and $k_{2\to0}$, if the bond is currently at State 2. Two random numbers, $\xi_i\left(i=1,2\right)$, uniformly distributed between 0 and 1, are generated. The durations needed for the random events to occur, $\tau_i\left(i=1,2\right)$, are obtained by solving

$$\int_0^{\tau_i} a_i\left(t\right)dt = \ln\left(1/\xi_i\right),\ i=1,2\,. \tag{S1}$$

The duration for the next random event to occur, Δt, is then given by

$$\Delta t = \min\left(\tau_i\right),\ i=1,2\,. \tag{S2}$$

The next random event is immediately determined. Subsequently, the bond state and time are updated. The above procedure will be looped until the bond is dissociated. .

Sometimes, a bond is already dissociated before the applied force reaches the clamped value in the simulation. When these short-lived events are excluded in averaging lifetime and timing starts from the time exactly when the bond is clamped and continues until the bond is dissociated, the corresponding mean lifetime is denoted as T_1. On the other hand, if these short-lived events are included in averaging lifetime and timing starts from the time

exactly when the force is applied and continues until the bond is dissociated, it is denoted as T_0. We compute 10000 lifetime for each force in order to get the mean lifetime and the error is 1%. The flow chart of simulation procedure is shown in Fig. S1.

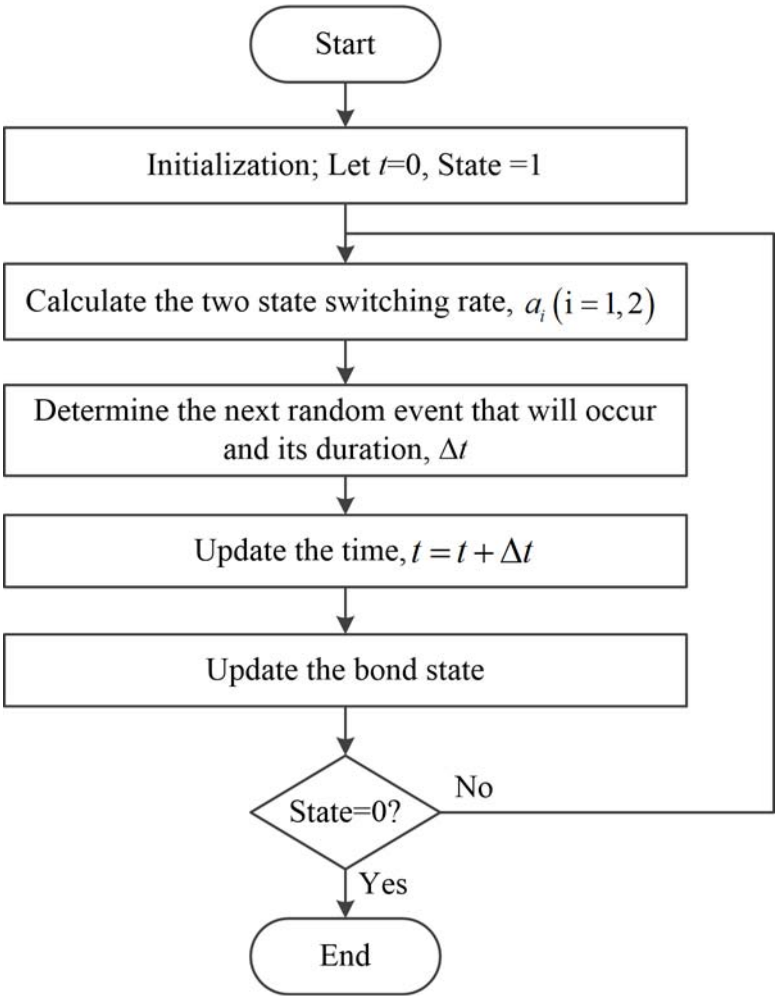

Figure S1 Flow chart of kinetic Monte Carlo method to simulate T_0 and T_1 for a single catch bond.
